# Diagnosis of Peritonsillar Abscess—A Prospective Study Comparing Clinical with CT Findings in 133 Consecutive Patients

**DOI:** 10.3390/diagnostics15020228

**Published:** 2025-01-20

**Authors:** François Voruz, Rebecca Revol, Christophe Combescure, Yan Monnier, Minerva Becker, Nicolas Dulguerov

**Affiliations:** 1 Department of Clinical Neurosciences, Clinic of Otorhinolaryngology—Head and Neck Surgery, Geneva University Hospitals, University of Geneva, 1211 Geneva, Switzerland; 2 Department of Health and Community Medicine, Geneva University Hospitals, University of Geneva, 1211 Geneva, Switzerland; 3 Diagnostic Department, Division of Radiology, Geneva University Hospitals, University of Geneva, 1211 Geneva, Switzerland

**Keywords:** peritonsillar abscess, tonsillar abscess, quinsy, trismus, contrast-enhanced computed tomography (CT), clinical assessment, diagnostic performance

## Abstract

**Background:** Peritonsillar abscess (PTA) is relatively common but challenging to diagnose clinically. Several clinical signs may be used, with unknown performances. We evaluated and compared the diagnostic performance of individual and combined clinical signs (*trismus*, *edema*, *pharynx immobility*, *uvula deviation*, *hot potato voice*, and *overall clinical impression*) assessed by an otolaryngologist and of contrast-enhanced computed tomography (CT) to detect acute PTA. **Methods:** Prospective study in 133 consecutive adult patients (77 males, mean age = 33 years) with suspected clinical PTA and CT obtained in the emergency setting of a tertiary care hospital between November 2020 and October 2022. The standard of reference consisted of surgically proven pus within 24 h of CT or a favorable clinical evolution at 48 h without surgical intervention. **Results:** PTA was present in 117/133 (88%) patients, with no difference between mean age or sex distribution between the groups with and without PTA. None of the evaluated clinical signs were associated with PTA (OR = 1.26–5.43, *p* > 0.05), whereas the CT finding “abscess” was significantly associated with PTA (OR = 67.2, *p* < 0.0001). The sensitivity of individual clinical signs varied between 19.7% and 73.5%, and the sensitivity of CT was significantly higher for all clinical signs (95.7%, *p* < 0.0001) except for *overall clinical impression* (97.4%, *p* = 0.7266). The specificity of clinical signs varied between 12.5% and 93.8%, and the specificity of CT was significantly higher (75%, *p* < 0.05) for *overall clinical impression* and *edema*. All clinical signs together yielded an area under the curve (AUC) = 0.677. **Conclusions:** In adults, clinical assessment alone using independent clinical signs and overall clinical impression does not allow a reliable diagnosis of PTA, even when performed by an otolaryngologist. CT is reliable in diagnosing PTA and, whenever available, should be the examination method of choice for diagnosing PTA, especially by a non-specialist.

## 1. Introduction

### 1.1. Epidemiology, Clinical Presentation, and Treatment

Peritonsillar abscess (PTA), also known as *quinsy*, is one of the most common—but potentially life-threatening—acute neck infections [1]. It is a collection of pus within the palatine tonsil or the peritonsillar space, a virtual anatomical space between the tonsillar capsule and the superior pharyngeal constrictor muscle. Among several pathophysiological hypotheses, PTA is classically regarded as the final stage of an infectious continuum that begins with tonsillitis, progresses to tonsillar cellulitis, and ultimately results in pus accumulation. Its etiopathogenesis is polybacterial, primarily attributed to *Streptococcus pyogenes* and *Fusobacterium necrophorum* [2,3,4]. Notably, minor sialadenitis has been proposed as a causative factor [5,6]. PTA may present as a co-diagnosis during infectious mononucleosis [1]. PTA primarily affects young adults [3,7], with a slight predominance in males [4,7,8,9,10], significantly impacting their quality of life [11]. Smoking is recognized as a well-established risk factor [7,12]. An estimation from 1995 suggested an incidence of 1/3300 person-years [13].

The symptomatology of PTA is evident, almost always characterized by severe lateralized odynophagia, with or without limited mouth opening (trismus), altered voice and speech, cervical lymphadenitis, restricted head movement, and a decline in general condition [8]. Patients often meet sepsis criteria [9]. Up to 10% of potentially life-threatening complications may arise [8], including Lemierre’s syndrome [14], contiguous spread into deeper neck spaces (typically parapharyngeal or retropharyngeal, and exceptionally submandibular [15] and buccal [16]), potentially leading to upper airway obstruction, mediastinitis, or necrotizing fasciitis [17]. The management of a PTA varies from center to center, as no clear evidence-based recommendations favor one approach over another. It typically involves antibiotic therapy along with repeated puncturing, or surgical drainage, or tonsillectomy.

### 1.2. Diagnosis

Since this condition is commonly encountered in daily emergency settings worldwide, diagnostic accuracy is crucial for the patient’s prognosis and relies on the experience of the emergency physician, as well as available resources. Accurately differentiating PTA from cellulitis may be challenging, with significant consequences for the patient. Following the Latin aphorism “Ubi pus, ibi evacua”, a collection of pus must be evacuated to ensure healing; therefore, mistakenly identifying a PTA as tonsillitis or tonsillar cellulitis can prolong suffering and create timely opportunities for potential complications. The best diagnostic method is debated; the literature provides insights into various diagnostic modalities, including straightforward and costless clinical judgment alone. Since the oropharynx is a symmetrical mobile soft structure, some signs are empirically recognized as good predictors of a PTA, such as limited mouth opening, asymmetry, bulging, decreased motion, and voice change, though their diagnostic performance remains uncertain [18,19]. Morphologic changes in neutrophils may help in predicting an infection [20] but do not differentiate cellulitis from a purulent abscess. Transcervical and transoral ultrasonography (US) have increasingly been proposed as accurate, safe, and cost-effective alternative diagnostic modalities, especially in children [21,22]. Nevertheless, physical limitations in inserting the oral US probe in patients with trismus and gag reflex, along with examiner dependence, are important drawbacks of transoral US [19]. Blind or echo-guided punctures have also been suggested as diagnostic [21] and therapeutic procedures [23,24]. However, these anxiogenic and painful interventions may be challenging due to trismus [23] or lack of cooperation. They can be unreliable for correctly locating the pus (especially if the abscess is multiloculated), leading to false negatives and multiple punctures that cause notable discomfort for the patient. Finally, accidental puncture of the internal carotid artery, sometimes tortuous and aberrant [25,26], could result in uncontrollable bleeding or hematoma [19]. Contrast-enhanced computed tomography (CT) is widely used in emergency departments for diagnosing PTA [19,27,28]. Additionally, CT enables the detection of multiloculated abscesses or vascular variants [25,26,29,30,31,32], which may complicate surgical treatment, thus aiding in surgical planning. Ultimately, only the direct visualization of pus during surgical exteriorization allows for a definitive diagnosis.

The ideal diagnostic method should be efficient, accurate, noninvasive whenever feasible, and cost-effective. Achieving all these properties in a single modality is challenging. This study aimed to evaluate the diagnostic performance of clinical examination alone and contrast-enhanced CT, comparing both assessments with the standard of reference, which consisted of intraoperative and follow-up findings 48 h after presentation. We selected these two modalities over others due to the previously mentioned limitations of US and puncture and because they are commonly available in most emergency centers and accessible to non-specialists.

## 2. Materials and Methods

### 2.1. Study Design

This was a single-center prospective study carried out in a cohort of consecutive patients seen in the emergency setting of a tertiary care hospital. Based on the decision of the ethics committee of our institution (Protocol No. 2020-01516), no informed consent was required for data analysis in the absence of specific intervention. Consequently, regarding patient and public involvement, the patients were not involved in the design, choice of outcomes measured, or recruitment of this study.

### 2.2. Population

Adult patients (≥18 years old) presenting with the clinical suspicion of a single-sided PTA, who had undergone clinical evaluation using five distinct signs and contrast-enhanced CT in an emergency setting, were included in this study between November 2020 and October 2022. Exclusion criteria were the following: life-threatening medical conditions, contraindications to CT, deep neck abscess extending to anatomical spaces other than tonsillar or peritonsillar (e.g., parapharyngeal, retropharyngeal), and pregnancy.

### 2.3. Study Protocol, Clinical Evaluation and CT Criteria, and Standard of Reference

**Figure 1:**
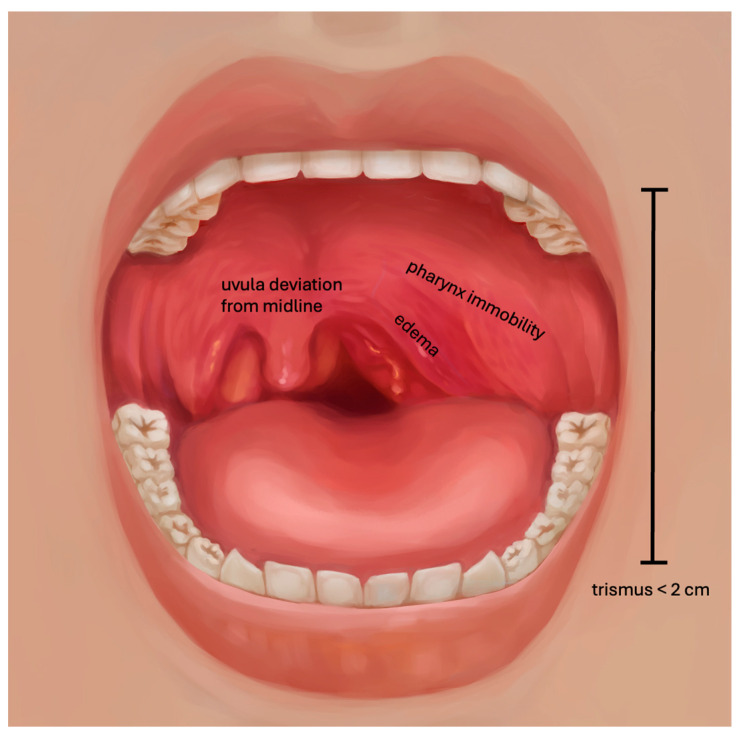
Clinical criteria used for peritonsillar abscess. *Hot potato voice* may be observed while vocalizing.

Patients presenting to the emergency department with severe sore throat were examined by a resident otolaryngologist in each case, assessing the presence or absence of five independent clinical signs: trismus, edema, pharynx immobility, uvula deviation, and hot potato voice (Figure 1). Trismus was defined as the inability to open the mouth wider than 2 cm between the upper and lower central teeth. Edema was defined as mucosal thickening of the tonsillar area. Pharynx immobility was defined as the absence of horizontal or vertical movement of the tonsillar area while vocalizing the open vowel [a] as would be pronounced in English (short “a”). Uvula deviation was defined as the shift of the uvula from the midline. Hot potato voice (or muffled voice) was defined by the lack of resonance of the voice while speaking. No scoring was made, but we assessed the overall clinical impression, which was defined as the examiner’s general clinical judgment that a PTA was present. Several otolaryngologists participated during the two-year recruitment period. Following the initial clinical examination confirming the suspicion of a PTA, each patient underwent a contrast-enhanced CT of the neck, as is standard practice. The CT examinations adhered to the As Low As Reasonably Acceptable (ALARA) and As Low As Diagnostically Acceptable (ALADA) principles, which are routinely applied in our institution. CT images were captured immediately after the intravenous injection of contrast material using the following parameters: 90 kV, 220–250 mA, single collimation width = 0.6 mm, and pitch factor = 0.8. The volumetric CT Dose Index (CTDIvol) per CT examination ranged from 8.9 to 9.3 mGy, while the corresponding Dose Length Product (DLP) ranged from 200 to 420 mGy·cm, depending on the patient’s morphology. These values fell below the established European diagnostic reference levels (DRLs) for CT examinations of the neck in suspected abscesses, which are 20 mGy and 500–600 mGy·cm, respectively [33,34,35].

**Figure 2:**
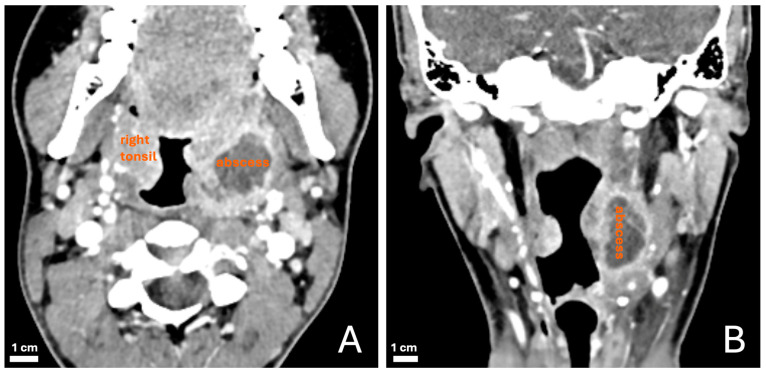
Radiological criteria used for peritonsillar abscess (PTA). Contrast-enhanced axial (**A**) and coronal (**B**) CT slices. Here, PTA is defined by fluid collection in the left peritonsillar space with serpiginous, irregular, and thick peripheral rim enhancement.

The CT scans were interpreted by the on-call radiologist prior to any intervention. This radiological report (real-world data) was used in the current study, and no further prospective or retrospective radiological interpretations were conducted. The CT criteria for a PTA included a low-attenuation (fluid) collection of ≥1 cm surrounded by a contrast-enhancing, irregular rim within the tonsil or the peritonsillar space, in accordance with the current literature (Figure 2). Following the CT of the neck, surgical exploration and treatment—both part of the gold standard—were performed within 24 h, mostly within 8 h. According to the standard of reference, a definitive PTA was defined as visible pus expressed during the surgical intervention (either immediate drainage under local anesthesia or immediate tonsillectomy under general anesthesia). If no radiological collection was detected on CT and the clinical evolution was favorable without surgery within 48 h, the definitive diagnosis of PTA was ruled out. Posterior (or retrotonsillar) PTA location was defined as an abscess lying posteriorly to the tonsil. Demographic and clinical data were obtained from patients’ charts.

### 2.4. Statistical Analysis

Calculating a formal sample size was challenging due to insufficient data. The sample size calculation relies on the prevalence of the PTA, the prevalence of the associated signs, and the magnitude of the association between the signs and PTA. Consequently, no sample size calculation was performed. The *t*-test and Chi-2 test were used to compare age and sex among subgroups of patients with and without PTA, as well as with and without posterior PTA location. The association between PTA and each separate clinical sign and *overall clinical impression* was assessed by odds ratios and tested with exact Fisher tests. The association between posterior PTA location with clinical signs and mean age was assessed with multivariate logistic regression. The diagnostic performance of clinical signs and CT in detecting PTA was assessed by means of sensitivity, specificity, accuracy, positive and negative predictive values (PPV and NPV), and positive and negative likelihood ratios (LR+ and LR−). The 95% confidence intervals (CIs) were calculated using the Clopper–Pearson exact method for proportions and the approach proposed by Simel et al. for likelihood ratios [36] (package DTComPair v1.2.2 for R [37]). To account for the paired design, comparisons with CT were performed using the exact McNemar test (package exact2x2 v1.6.5 for R [38]) for sensitivities, specificities, and accuracies; a regression model approach for the likelihood ratios; and the method proposed by Moskowitz et al. for the predictive values [39] (package DTComPair v1.2.2 for R [37]). The diagnostic performance of the number of clinical signs per patient was assessed by a Receiver Operating Characteristic (ROC) curve approach and its area under the curve (AUC) [40]. All statistical analyses were conducted with R version 4.0.2 (R Core Team, 2021). R: A language and environment for statistical computing. R Foundation for Statistical Computing, Vienna, Austria. (https://www.R-project.org (accessed on 11 March 2024)). Statistical tests were two-sided with a significance level of 0.05. Our dataset was free of missing values.

## 3. Results

**Figure 3:**
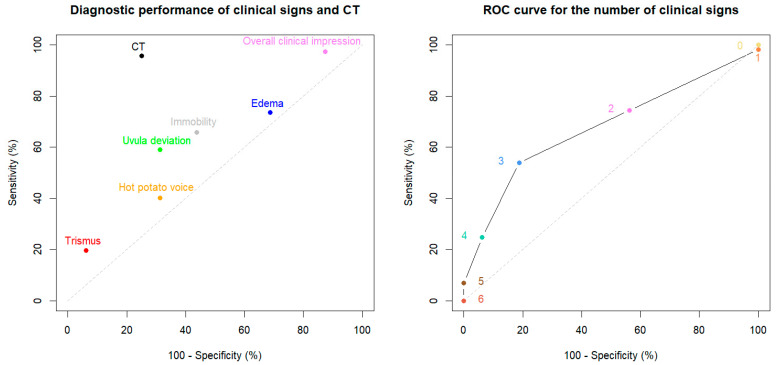
Diagnostic performance expressed as sensitivity–specificity pairs for each individual clinical sign, *overall clinical impression*, and CT, respectively (**left** panel), and Receiver Operating Characteristic (ROC) curve for the number of clinical signs (**right** panel). Numbers shown on the ROC curve (**right** panel) are the thresholds: the sensitivities are the proportions of patients with peritonsillar abscess (PTA) presenting a number of signs greater or equal to the threshold, and the specificities are the proportions of patients without PTA presenting a number of signs lower than the threshold.

**Figure 4:**
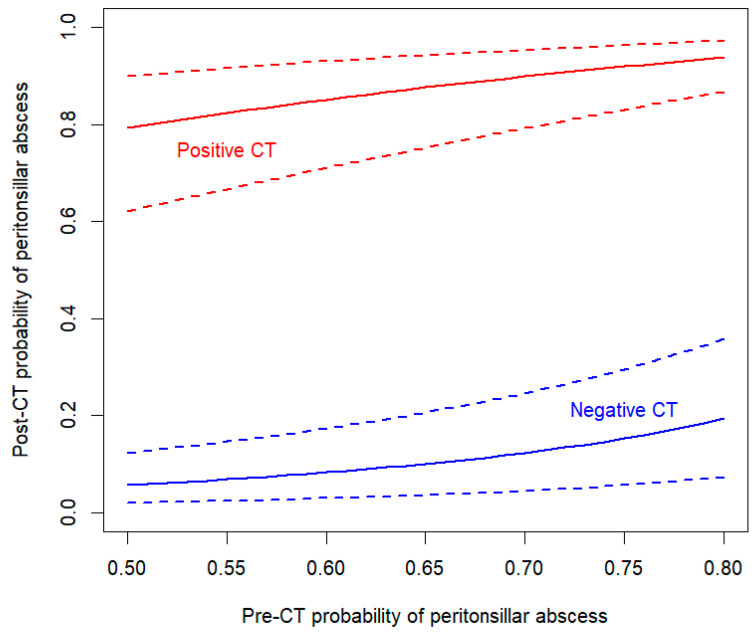
Post-CT probability of peritonsillar abscess (PTA) as a function of pre-CT probability when the CT is positive (solid red curve) and when the CT is negative (solid blue curve). These curves show how the probability of PTA is modified by the CT result. Overall, when the CT is negative (respectively, positive), the probability decreases (respectively, increases) importantly. The dashed curves represent the 95% confidence intervals of the post-CT probability of PTA.

A total of 133 adult patients with suspected PTA were included, for whom Table 1 summarizes the pertinent demographic, CT, and PTA characteristics. Based on the standard of reference, 117/133 (88%) patients had PTA, while 17/133 (12%) did not. No differences in sex (*p* = 0.2) or mean age (*p* = 0.6) were found between patients with and without PTA. Posterior location was present in 33/117 (28%) cases of PTA. No difference in sex (*p* = 0.1) or mean age (*p* = 0.3) was found between patients with posterior location and other locations. Among the five clinical signs studied, only *edema* was associated with posterior location (OR = 3.3 (95%CI 1 to 10), *p* = 0.04). Complications (bilaterality or extension to neck spaces beyond the tonsillar and peritonsillar regions) were detected in 6/116 (5%) of abscess-positive CT exams. In contrast to CT, none of the clinical signs were associated with PTA (Table 2). The diagnostic performance of each clinical sign, *overall clinical impression*, and CT are detailed in Table 3 and graphically represented in Figure 3, respectively. The AUC for the number of signs was 0.677 (95%CI 0.559 to 0.795), as represented in Figure 3. The post-CT probability of PTA as a function of pre-CT probability is illustrated in Figure 4.

The following results are included in the Supplementary Materials: accuracy of clinical signs compared to the accuracy of CT (Table S1), PPV, and NPV of clinical signs compared to the respective CT values (Table S2), proportion of patients according to the number of clinical signs (Table S3), specificity and sensitivity based on the number of clinical signs (Table S4), and LR of clinical signs compared to the LR of CT (Table S5).

## 4. Discussion

### 4.1. Diagnosis

None of the five clinical signs evaluated independently in this study were reliable enough to accurately diagnose PTA; none, including the *overall clinical impression*, had a significant association with PTA (Table 2), and all clinical signs performed worse diagnostically compared to CT (Table 3). The sensitivity of each specific clinical sign ranged from 20% to 66%, without even getting close to the sensitivity of CT. Only one sign, *trismus*, exhibited slightly higher specificity than CT, but this result was not statistically significant. *Edema* showed a significantly lower specificity than CT. While the presence of *edema* increased the odds of a posterior PTA location, it emerged as the least effective sign for diagnosing PTA. Due to the risk of upper airway obstruction, *edema* must still be clinically assessed in all patients despite its unreliability in diagnosing PTA. Although *trismus*, *pharynx immobility*, *uvula deviation*, and *hot potato voice* had a comparable PPV to CT (Table S2), the present study found no significant association between these clinical signs and PTA, raising doubts about their utility. Additionally, trismus may be overestimated in the clinical practice and literature, as a pain-driven slight limitation in mouth opening can occur in conditions like pharyngitis, tonsillitis, or tonsillar cellulitis. We applied conservative criteria to define true trismus, which arises from mechanical impairment of the pterygoid muscle area due to the presence of pus and inflammation rather than solely from pain.

The *overall clinical impression* was very sensitive; however, its lack of specificity and low LR+ did not prove useful in diagnosing PTA. This supports the findings of another study, which concluded that clinical impression alone was unreliable for diagnosing PTA [19]. Nevertheless, due to its availability, the *overall clinical impression* could still serve as an initial screening tool, aiding in early PTA detection and reducing diagnostic delays, especially in resource-limited countries. However, low specificity resulted in high false-positive rates, potentially leading to unnecessary surgical procedures and strain on resources. False positives may also result in economic losses and reduced trust in a healthcare system. To maximize its benefits, the *overall clinical impression* could be part of a tiered diagnostic strategy, with the first tier using *overall clinical impression* (low-specificity test) to identify individuals at risk for PTA, followed by confirmatory testing with CT. The five clinical signs examined independently were neither reliable nor useful for diagnosing PTA. In most cases of PTA, two or more clinical signs were present. Nevertheless, incrementing positive clinical signs did not yield acceptable diagnostic performance for diagnosing PTA (Figure 3). This finding may reflect the daily challenge of clinical determination based solely on clinical assessment.

CT only lowered the pre-test PTA probability, having a moderately high NPV and a fair LR− (Figure 4, Tables S2 and S5). In cases of clinically suspected PTA, the presence of a contrast-enhanced fluid collection on CT was a reliable indicator, suggesting the presence of an abscess. False-negative CT readings occurred in only 4.3% of cases; false negativity in the context of PTA results in delayed appropriate treatment and increased risk of complications. However, scanning every suspected PTA could potentially worsen the clinical impression. A retrospective study of 362 patients found that those who underwent CT were more likely to be hospitalized and require surgical intervention rather than bedside drainage [41]. One could hypothesize that CT might create a more severe impression than clinical assessment alone, influencing the clinician’s decision toward surgery. Furthermore, CT incurs costs and may contribute to potential harm associated with radiation exposure and/or the administration of intravenous contrast material, the latter being necessary for diagnosis. Due to increased awareness of radiation safety, DRLs for CT examinations have evolved over the past few years, reflecting technological advancements and the optimization of acquisition protocols. The development of multi-detector CT scanners and dose reduction technologies has led to the establishment of lower DRLs, promoting safer imaging practices [33,42]. Recent studies advocate for DRLs tailored to specific clinical indications, acknowledging that different clinical questions may require different imaging protocols [35]. As mentioned in the Section 2, the CT protocol used in this study yielded lower CTDIvol and DLP values than the current established DRLs. However, due to significant variations in DRLs across different institutions and countries, an international collaboration to standardize radiation doses in CT is needed in the future [43]. Ultimately, obtaining urgent CT may delay treatment [41] in patients who meet the criteria for sepsis, experience severe pain, or may be at risk for rapid upper airway obstruction. Since only 5% of complications were identified on imaging in this study, routinely performing CT for every suspected case of PTA to proactively detect deep neck space extension, vascular anomalies, or contralateral involvement is not advisable due to concerns over radiation exposure, potential treatment delays, and significant costs. Therefore, a systematic CT in the initial evaluation of suspected PTA should be reserved for patients with ambiguous clinical presentations, suspected complications (e.g., spread into the parapharyngeal or retropharyngeal spaces), bilateral PTA, or when a comprehensive clinical examination cannot be conducted (e.g., in cases of severe trismus), as indicated in the literature [1].

Although this study did not find utility in differentiating tonsillar cellulitis from PTA, a thorough clinical assessment is still mandatory to confirm a high suspicion of PTA and rule out the risk of immediate upper airway obstruction. Clinical assessment is noninvasive, quick (taking less than a minute), economical, and accessible to non-specialists. The *overall clinical impression* aids in selecting patients, while CT is even better for confirming the attitude. This is particularly relevant in non-specialist settings, unlike this study, where the clinical assessment, even when conducted by an otolaryngologist, failed to show an association with PTA. Therefore, CT is valuable for addressing inter-individual variability in clinical experience. If available, a CT should be considered the examination method of choice for diagnosing PTA by a non-specialist.

### 4.2. Strengths and Limitations

This real-life population featured a balanced sex ratio and age distribution, aligning well with the existing literature [4,7,8,9]. Conducted as a prospective study, we implemented strict inclusion criteria along with a clearly defined standard of reference.

While we did not evaluate the reproducibility of our radiological and clinical assessments, they were performed under paired supervision (training physician and attending physician), which is standard practice at our hospital. This approach minimizes the likelihood of unchecked cognitive bias from a single-person assessment and reduces the risk of errors due to insufficient external validation.

Our population presenting in emergency situations with suspected PTA had a high prevalence of PTA; therefore, few patients without PTA were included in this cohort. This limitation affected the statistical analysis but simultaneously represented real-life data relevant to our clinical question. With this sample of patients, the power of our study was 80%, allowing for the detection of only strong associations with PTA (odds ratio > 5 for a prevalence of 50 to 70% and odds ratio > 10 for a prevalence of 25% or less).

The delay between imaging and pus drainage was not evaluated in this study. Drainage could either be performed through an incision under local anesthesia or through a tonsillectomy under general anesthesia. While a simple incision is much quicker, the delay between CT and tonsillectomy typically ranges from 2 to 8 h, depending on the availability of the operating room. These varying time periods may have influenced some data, such as the association between clinical signs and the presence of pus at different time points.

## 5. Conclusions

In adults, clinical assessment alone does not allow for a reliable diagnosis of PTA, even when performed by an otolaryngologist. Although the overall clinical impression was very sensitive, its lack of specificity did not prove helpful in diagnosing PTA. Contrast-enhanced CT is very utile and reliable in diagnosing PTA and should be used by the non-specialist when available. For the specialist, the overall clinical impression could be part of a tiered diagnostic strategy, with the first tier using overall clinical impression to identify individuals at risk for PTA (high sensitivity), followed by confirmatory testing with CT (improved specificity).

## Figures and Tables

**Table 1:** Demographic, CT, and abscess characteristics.

Total number of included patients	133
Mean age—yr (range)	33 (18–78)
Male—No. (%)	77 (58)
CT—No. of patients (%)	133 (100)
Radiological collection	116 (87)
Unilateral	115 (86)
Complicated *	6 (5)
No radiological collection	17 (13)
Peritonsillar abscess (pus)—No. (%)	117 (88)
Right	52 (44)
Left	66 (56)
Posterior location	33 (28)
Other location	84 (72)
No clinical sign	2 (2)
1 clinical sign only	28 (24)
≥2 clinical signs	87 (74)

*: bilateral or extended beyond peritonsillar space.

**Table 2:** Test data and odds ratios for the association with peritonsillar abscess (PTA).

	True Positive *	True Negative *	False Positive *	False Negative *	OR (95%CI) **	*p*-Value ***
CT	112	12	4	5	67.2 (15.87 to 284.57)	<0.0001
Trismus	23	15	1	94	3.67 (0.46 to 29.23)	0.3026
Edema	86	5	11	31	1.26 (0.41 to 3.92)	0.7655
Pharynx immobility	77	9	7	40	2.48 (0.86 to 7.14)	0.1021
Uvula deviation	69	11	5	48	3.16 (1.03 to 9.69)	0.0581
Hot potato voice	47	11	5	70	1.48 (0.48 to 4.53)	0.5915
Overall clinical impression	114	2	14	3	5.43 (0.83 to 35.34)	0.1096

OR: odds ratio. *: true positive = patients with PTA at surgery (pus) and collection at CT or PTA at clinical evaluation; true negative = patients without PTA at surgery (no pus) or with favorable clinical evolution at 48 h and without collection at CT or without PTA at clinical evaluation; false negative = patients with PTA at surgery (pus) and no collection at CT or no PTA at clinical evaluation; false positive = patients without PTA at surgery (pus) but with collection at CT or PTA at clinical evaluation. **: OR > 1 means that the clinical sign is more frequent in patients with PTA than in patients without PTA. ***: exact Fisher’s test.

**Table 3:** Diagnostic performance of individual clinical signs and *overall clinical impression* compared with CT, for detecting peritonsillar abscess.

	Sensitivity			Specificity		
	n/N	Sen (95%CI)	*p*-Value *	n/N	Spe (95%CI)	*p*-Value *
CT	112/117	95.7 (90.3 to 98.6)		12/16	75.0 (47.6 to 92.7)	
Trismus	23/117	19.7 (12.9 to 28.0)	<0.0001	15/16	93.8 (69.8 to 99.8)	0.3750
Edema	86/117	73.5 (64.5 to 81.2)	<0.0001	5/16	31.2 (11.0 to 58.7)	0.0391
Pharynx immobility	77/117	65.8 (56.5 to 74.3)	<0.0001	9/16	56.2 (29.9 to 80.2)	0.3750
Uvula deviation	69/117	59.0 (49.5 to 68.0)	<0.0001	11/16	68.8 (41.3 to 89.0)	>0.99
Hot potato voice	47/117	40.2 (31.2 to 49.6)	<0.0001	11/16	68.8 (41.3 to 89.0)	>0.99
Overall clinical impression	114/117	97.4 (92.7 to 99.5)	0.7266	2/16	12.5 (1.6 to 38.3)	0.0020

*: *p*-values refer to comparison with CT.

## Data Availability

The raw data supporting the conclusions of this article will be made available by the authors on request.

## Supplementary Materials

The following supporting information can be downloaded at: https://www.mdpi.com/article/10.3390/diagnostics15020228/s1, Table S1: Accuracy (i.e., proportion of patients correctly classified as positive or negative) of individual clinical signs, *overall clinical impression*, and CT for the detection of peritonsillar abscess (PTA). Table S2: Positive and negative predictive values (PPVs) of individual clinical signs, *overall clinical impression*, and CT for the detection of PTA. Table S3: Proportion of patients with PTA based on the number of clinical signs. Table S4: Specificity (Spe) and sensitivity (Sen) based on the number of clinical signs present at evaluation. Table S5: Likelihood ratios (LRs) of individual clinical signs, *overall clinical impression*, and CT for the detection of PTA.
