# Supplementary material for: Diagnosis of Peritonsillar Abscess—A Prospective Study Comparing Clinical with CT Findings in 133 Consecutive Patients
Source: Diagnostics (Basel). 2025 Jan 20;15(2):228. doi: 10.3390/diagnostics15020228 (PMC11765337; doi:10.3390/diagnostics15020228)
Supplement: Supplementary file 1 [file diagnostics-15-00228-s001.zip › diagnostics-3392462-supplementary.pdf]

SUPPLEMENTARY MATERIALS

Table S1: Accuracy (i.e., proportion of patients correctly classified as positive or negative) of individual clinical signs and *overall clinical impression* compared with CT, for detecting peritonsillar abscess.

	**Accuracy**		
	**n/N**	**Acc (95%CI)**	***P*-value***
CT	124/133	93.2 (87.5 to 96.9)	
Trismus	38/133	28.6 (21.1 to 37.0)	<0.0001
Edema	91/133	68.4 (59.8 to 76.2)	<0.0001
Pharynx immobility	86/133	64.7 (55.9 to 72.7)	<0.0001
Uvula deviation	80/133	60.2 (51.3 to 68.5)	<0.0001
Hot potato voice	58/133	43.6 (35.0 to 52.5)	<0.0001
Overall clinical impression	116/133	87.2 (80.3 to 92.4)	0.0963

*: p values refer to comparison with CT

Table S2: Positive and negative predictive values (PPV and NPV) of individual clinical signs and *overall clinical impression* compared with CT, for detecting peritonsillar abscess.

	**Positive predictive value**			**Negative predictive value**		
	**n/N**	**PPV (95%CI)**	***P*-value***	**n/N**	**NPV (95%CI)**	***P*-value***
CT	112/116	96.6 (91.4 to 99.1)		12/17	70.6 (44.0 to 89.7)	
Trismus	23/24	95.8 (78.9 to 99.9)	0.8704	15/109	13.8 (7.9 to 21.7)	<0.0001
Edema	86/97	88.7 (80.6 to 94.2)	0.0078	5/36	13.9 (4.7 to 29.5)	<0.0001
Pharynx immobility	77/84	91.7 (83.6 to 96.6)	0.0538	9/49	18.4 (8.8 to 32.0)	<0.0001
Uvula deviation	69/74	93.2 (84.9 to 97.8)	0.2735	11/59	18.6 (9.7 to 30.9)	<0.0001
Hot potato voice	47/52	90.4 (79.0 to 96.8)	0.1686	11/81	13.6 (7.0 to 23.0)	<0.0001
Overall clinical impression	114/128	89.1 (82.3 to 93.9)	0.0017	2/5	40.0 (5.3 to 85.3)	0.295

*: p values refer to comparison with CT

Table S3: Proportion of patients with a peritonsillar abscess according to the number of clinical signs.

**Nb signs**	**Peritonsillar abscess, n/N (%)**
0	2/2 (100.0 %)
1	28/35 (80.0 %)
2	24/30 (80.0 %)
3	34/36 (94.4 %)
4	21/22 (95.5 %)
5	8/8 (100.0 %)

Table S4: Specificity (Spe) and sensitivity (Sen) based on the number of clinical signs present at evaluation, for detecting peritonsillar abscess.

Signs	Spe (95%CI)	Sen (95%CI)
≥1	0.0 (0.0 to 20.6)	98.3 (94.0 to 99.8)
≥2	43.8 (19.8 to 70.1)	74.4 (65.5 to 82.0)
≥3	81.3 (54.4 to 96.0)	53.8 (44.4 to 63.1)
≥4	93.8 (69.8 to 99.8)	24.8 (17.3 to 33.6)
≥5	100.0 (79.4 to 100.0)	6.8 (3.0 to 13.0)

Table S5: Likelihood ratios of clinical signs and *overall clinical impression* compared with CT, for detecting peritonsillar abscess.

	LR+ (95%CI)	*P*-value*	LR- (95%CI)	*P*-value*
CT	3.83 (1.64 to 8.95)		0.06 (0.02 to 0.14)	
Trismus	3.15 (0.46 to 21.73)	0.8622	0.86 (0.73 to 1.00)	<0.0001
Edema	1.07 (0.76 to 1.51)	0.0051	0.85 (0.39 to 1.86)	<0.0001
Pharynx immobility	1.50 (0.85 to 2.66)	0.0291	0.61 (0.37 to 1.00)	<0.0001
Uvula deviation	1.89 (0.90 to 3.96)	0.2357	0.60 (0.40 to 0.89)	<0.0001
Hot potato voice	1.29 (0.60 to 2.75)	0.1089	0.87 (0.61 to 1.25)	<0.0001
Overall clinical impression	1.11 (0.92 to 1.34)	0.0035	0.21 (0.04 to 1.14)	0.1888

*: *p*-values refer to comparison with CT
LR+: positive likelihood ratio
LR-: negative likelihood ratio
